# A comprehensive assessment of urinary iodine concentration and thyroid hormones in New Zealand schoolchildren: a cross-sectional study

**DOI:** 10.1186/1475-2891-11-31

**Published:** 2012-05-08

**Authors:** Sheila A Skeaff, Christine D Thomson, Noela Wilson, Winsome R Parnell

**Affiliations:** 1 Department of Human Nutrition, University of Otago, Dunedin, 9054, New Zealand; 2 LINZ Activity Unit, University of Otago, Dunedin, 9054, New Zealand

**Keywords:** Iodine, Iodine deficiency, Urinary iodine concentration, Children, Thyroid hormones

## Abstract

**Background:**

Insufficient iodine in children’s diets is of concern because thyroid hormones are needed for normal growth and development, particularly of the brain. This study aimed to carry out a comprehensive assessment of the iodine status of New Zealand schoolchildren using a range of biochemical indices suitable for populations (i.e. urinary iodine concentration) and individuals (i.e. thyroid hormones).

**Methods:**

The New Zealand National Children’s Nutrition Survey was a cross­‒sectional survey of a representative sample of schoolchildren aged 5­‒14 years. Children were asked to provide a casual urine sample for the determination of urinary iodine concentration (UIC) and a blood sample for the determination of thyroglobulin (Tg), Thyroid Stimulating Hormone (TSH), free thyroxine (fT4) and free triiodothyronine (fT3).

**Results:**

The median UIC was 68 μg/L (n = 1153), which falls between 50­‒99 μg/L indicative of mild iodine deficiency. Furthermore, 29% of children had an UIC <50 μg/L and 82% had an UIC <100 μg/L. The median Tg concentration was 12.9 μg/L, which also falls between 10.0­‒19.9 μg/L indicative of mild iodine deficiency. The Tg concentration of children with an UIC <100 μg/L was 13.9 μg/L, higher than the 10.3 μg/L in children with an UIC >100 μg/L (*P* = 0.001). The mean TSH (1.7 mU/L), fT4 (14.9 pmol/L), and fT3 (6.0 pmol/L) concentrations for these mildly iodine deficient New Zealand children fell within normal reference ranges.

**Conclusions:**

The UIC and Tg concentration indicate that New Zealand schoolchildren were mildly iodine deficient according to WHO/UNICEF/ICCIDD, and both are suitable indices to assess iodine status in populations or groups. The normal concentrations of TSH, fT4 and fT3 of these children suggest that these thyroid hormones are not useful indices of mild iodine deficiency.

## Background

New Zealand has a long history of iodine deficiency, with endemic goiter first reported in the 1920s. This is not surprising given that New Zealand soils are low in iodine and, despite being surrounded by the ocean, the typical diet contains small quantities of fish and other seafood. It was the work of Hercus and Purves who compared the median urinary iodine excretion from nine goitrous regions of New Zealand (i.e. 24‒62 μg/day) with non-­goitrous parts of Australia (i.e. 80‒147 μg/day) and the Pacific Island of Western Samoa (i.e. 172 μg/day), which finally prompted the iodization of table salt to 50 mg/kg in 1939 [1]. By the 1950s, the incidence of goiter had fallen from 20‒30% to less than one percent [2]. Because iodized salt was not used in manufactured foods, the regular consumption of dairy products (contaminated by iodophors used in milk processing) and iodized salt added at the table and in cooking is believed to be responsible for the adequate iodine intakes reported in New Zealand between the 1960s and the 1980s with urinary iodine excretion ranging from 120‒220 μg/day [3-6].

Iodine deficiency is defined by the World Health Organization (WHO) as a population median urinary iodine concentration (UIC) that falls below 100 μg/L, while a median UIC of 50–99 μg/L, 20-49 μg/L, and <20 μg/L indicates mild, moderate, and severe iodine deficiency, respectively [7]. Mild iodine deficiency has re‒emerged in Australia [8], and most recently, the United Kingdom [9], caused by a change in food patterns. In the 1990s there were reports in New Zealand of iodine deficiency in volunteer samples of adults [10-12] and in children aged 8‒10 years (yr) living in two cities [13]. A lack of iodine in the diets of children is of concern, as sufficient quantities of thyroid hormones are needed for normal growth and development, particularly of the brain.

The measurement of the UIC in a single or casual urine sample is the most commonly used index to assess iodine status in schoolchildren. In addition, blood constituents such as thyroid stimulating hormone (TSH), thyroglobulin (Tg), thyroxine (T4), and tri‒iodothyronine (T3) can also be measured. Severe to moderate iodine deficiency lowers circulating T4 concentrations, resulting in a subsequent rise in TSH. If iodine deficiency becomes severe enough, a decrease in T3 concentration may also be seen. Although a median UIC between 50–100 μg/L is used to classify mild iodine deficiency, the thyroid hormone concentrations of such populations typically fall within normal reference ranges. There is growing evidence that Tg is a more sensitive index of mild iodine deficiency than TSH, T4, and T3 [14-16], however, Tg is not routinely measured in cross‒sectional surveys. The aim of this study was to assess the iodine status of a representative sample of New Zealand schoolchildren using a range of biochemical indices.

## Subjects and methods

The Children’s Nutrition Survey was a cross‒sectional survey of a nationally representative sample of New Zealand school children aged 5‒14 year conducted between March and December 2002. Children were categorised into three ethnic groups based on parental report; Māori, Pacific, and New Zealand European & all Other ethnicities (NZEO). A two‒stage school‒based sampling frame was used to recruit children with over‒sampling of Māori and Pacific children to allow for ethnic specific analysis. In brief, schools were randomly selected from the Ministry of Education rolls and children from these schools were randomly selected in proportion to the number of children on the school roll. Socio‒demographic data, height, weight, and a 24‒hr diet recall were collected from participating children; because the iodine content of foods was neither complete nor reliable in the New Zealand Food Composition Database, iodine intakes were not determined. Full ethical approval was obtained from 13 regional health ethics committees. Informed written consent was obtained from parents or guardians of the children. Further details of the survey methodology and a summary of the key results can be found elsewhere [17]. Blood and urine samples were collected from a subset of urban children who agreed and whose parents consented to the sampling; children at the 66 rural schools were not included due to concerns that the length of time required to transport samples to the laboratory would affect blood stability. Sampling weights were based on the provision of a blood sample and not on participation in the survey. Non‒fasting venous blood samples were drawn into vacutainers (Becton Dickinson, Frankton Lakes, NJ, USA), refrigerated immediately after collection, and couriered to a central laboratory for processing the following morning. After centrifugation, serum and plasma samples were stored at −80°C until analysis. Casual or spot urine samples were collected from children during school hours, refrigerated, and couriered to the Department of Human Nutrition, University of Otago, the next day. Urine samples were stored at ‒20°C until analysis.

UIC was determined using a modification of the method of Pino et al. [18] at the Department of Human Nutrition, University of Otago. An external reference standard (Seronorm Trace Elements Urine, Sero As, Norway) was analysed with each batch of samples giving a mean iodine concentration of 93 μg/L (expected: 97 μg/L) with a coefficient of variation (CV) of 8.4% (n = 213).

Serum TSH was determined by Southern Community Laboratories using an ADVIA Centaur two-site sandwich immunoassay (Bayer Health Care Diagnostika, Vienna, Austria). The reference range was 0.10‒150 mU/L. Analysis of a supplied external reference standard (Ligand Plus) resulted in a CV of 5.1% (n = 291). Serum Tg concentration was determined using a DiaSorin radioimmunoassay (DiaSorin, Saluggia, Italy) in the Department of Human Nutrition, University of Otago. The reference range was 0.3‒500 μg/L. Analysis of a pooled internal control gave a CV of 8.5% (n = 20). Because these serum samples had been used to assess other nutrients prior to iodine, we measured the effect of up to four freeze‒thaw cycles on serum TSH and Tg and found there were no differences (n = 18).

Plasma free T4 (fT4) and free T3 (fT3) concentrations were determined by Southern Community Laboratories using an ADVIA Centaur fT4 and fT3, respectively, competitive chemiluminescent immunoassay (Bayer Health Care Diagnostika, Vienna, Austria). Plasma was used to determine fT4 and fT3 concentrations as there was insufficient serum for analysis of all four blood constituents (i.e. Tg, TSH, fT4 and fT3). Prior to analysis for this study, we tested the recovery of fT4 and fT3 in plasma, which was 100‒106% and 97‒107%, respectively, compared to serum values obtained from the same subjects. The range of detection of the fT4 assay was 1.3‒155 pmol/L and that of the fT3 assay was 0.3‒30.8 pmol/L. Analysis of an external reference standard (Ligand Plus) resulted in a CV of 8.1% (n = 261) and 5.7% (n = 297) for fT4 and fT3 assays, respectively.

All statistical analyses were carried out using the statistical package STATA 11.1 (StataCorp LP. College Station, TX, USA). We used survey commands to account for the survey design, weighting, and clustering. Socioeconomic status was determined by grouping children using the New Zealand Index of Deprivation scale, an index based on the residential address of the child [19]. The scale is divided into five quintiles, with quintile 1 representing the least deprived area and quintile 5 the most deprived area. Children allocated to quintile 1 and 2 were categorized as high socioeconomic status, children allocated to quintile 3 and 4 were categorized as medium socioeconomic status, and children allocated to quintile 5 as low socioeconomic status. Log transformation was carried out for data that was not normally distributed (i.e. UIC, TSH and Tg data) and geometric means reported. Linear regression analysis was used to determine the effect of age (5‒7 yr, 8‒10 yr, 11‒14 yr), sex (boy, girl), ethnicity (Māori, Pacific, NZEO), socioeconomic status (high, medium, low), and UIC (<50 μg/L and <100 μg/L) on Tg, TSH, fT4, and fT3. Bonferroni adjusted *P*‒values are reported for post‒hoc comparisons. All tests were two‒sided and the level of significance set at *P* < 0.05.

## Results

Two hundred schools were asked to participate in the survey and 172 agreed; of the 4728 children recruited, 3275 participated. A urine sample was collected from 1796 children and a blood sample from 1927 children. Serum samples were used to determine the concentration of a number of other nutrients (i.e. 25‒hydroxyvitamin D, selenium) prior to the analysis of the thyroid hormones; surplus serum or plasma was available for complete thyroid hormone (i.e. TSH, Tg, fT4 and fT3) analysis from 1212 children. The Deprivation Index (i.e. socioeconomic status) of 59 children was not known and these children were excluded from the analysis, resulting in a final sample size of 1153 children for which both urinary iodine and thyroid hormone data are available. There were no statistically significant differences between those children who gave a blood sample and those who did not by sex, age, socioeconomic status, and ethnicity.

The median (25th, 75th percentile) UIC of New Zealand children was 68 (50, 95) μg/L (Table 1), which falls within the range 50–99 μg/L that WHO/UNICEF/ICCIDD categorize as mild odine deficiency [7]. Furthermore, 29% of children had a UIC <50 μg/L and 82% had UIC <100 μg/L, above than the 20% and 50%, respectively, recommended by WHO/UNICEF/ICCIDD [7]. UIC did not differ by sex, age, socioeconomic status, or ethnicity. The median (25th, 75th percentile) Tg concentration for New Zealand children was 12.9 (8.1, 19.9) μg/L, which falls between 10.0-19.9 μg/L also indicative of mild iodine deficiency according to WHO/UNICEF/ICCIDD (Table 1) [7]. Tg concentration did not differ by sex, socioeconomic status, or ethnicity (Table 2). There was a significant effect of age, such that increasing age category decreased Tg concentration by 14% (*P* < 0.001). Children 5‒7 yr had a higher (geometric) mean Tg concentration than children 11‒14 yr (16.3 vs 11.2 μg/L; *P* = 0.002) and children 8‒10 yr had a higher (geometric) mean than children 11‒14 yr (13.8 vs 11.2 μg/L; *P* = 0.031); there was no significant difference in the (geometric) mean Tg concentration between 5‒7 yr and 8‒10 yr children. The concentration of Tg at all percentiles was higher in children 5‒7 yr compared to older children (Table 3). The (geometric) mean Tg of children with a UIC <50 μg/L was significantly higher than in children with an UIC ≥50 μg/L (15.6 vs 12.3 μg/L; *P* = 0.001) (Table 4). The (geometric) mean Tg of children with an UIC <100 μg/L was significantly higher than in children with an UIC ≥100 μg/L (13.9 vs 10.3 μg/L; *P* < 0.001) (Table 4). The (geometric) mean (95% Confidence Interval (CI)) TSH concentration of the children was 1.7 (1.6‒1.8) mU/L (Table 1). There were no significant differences in TSH concentration with regard to sex, age, socioeconomic category, or ethnicity (Table 2). There were also no differences in TSH concentration between children with an UIC <50 μg/L or UIC <100 μg/L (Table 4).

**Table 1:** Urinary iodine (UIC), Thyroglobulin (Tg), TSH, free T4, and free T3 concentration by sex, age, socioeconomic status, and ethnicity in New Zealand children

		**UIC (μg/L)**		**Tg (μg/L)**		**TSH (mu/L)**		**Free T4 (pmol/L)**		**Free T3 (pmol/L)**	
	**n**	**Median**	**25th,75th**	**Median**	**25th,75th**	**Mean**	**95% Cl**	**Mean**	**95% Cl**	**Mean**	**95% Cl**
Total	1153	68	50,95	12.9	8.1,19.9	1.7	1.6,1.8	14.9	14.6,15.2	6.0	5.9,6.1
Sex											
Male	611	69	50,94	12.6	17.9,19.2	1.7	1.6,1.8	14.8	14.5,15.1	6.0	5.9,6.0
Female	542	67	55,96	13.4	8.5,20.5	1.7	1.6,1.9	15.0	14.5,15.4	6.0	5.9,6.1
Age											
5-7 yr	315	63	50,88	16.2	10.4,24.2	1.6	1.5,1.8	15.4	14.9,15.8	6.0	5.9,6.1
8-10 yr	438	67	50,94	12.5	8.0,19.0	1.8	1.7,1.9	15.0	14.6,15.5	6.0	5.9,6.1
11-14 yr	400	75	51,106	11.1	7.4,16.4	1.7	1.6,1.8	14.4	13.9, 14.9	5.9	5.9,6.1
SES											
Low	567	70	50,98	12.8	8.0,19.9	1.7	1.6,1.8	15.2	14.7,15.7	6.0	5.9,6.2
Medium	347	68	50,93	12.4	8.0,20.4	1.6	1.5,1.7	15.0	14.5,15.5	5.9	5.9,6.0
High	239	66	50,93	13.2	9.0,19.2	1.8	1.7,1.9	14.6	14.1,15.1	6.0	5.9,6.2
Ethnicity											
NZEO	333	66	50,93	13.5	8.8,21.2	1.7	1.6,1.8	14.6	14.3,15.0	6.0	5.9,6.1
Māori	338	67	50,87	13.0	8.3,20.1	1.6	1.5,1.8	15.4	14.9,15.9	6.0	5.9,6.1
Pacific	482	73	50,105	12.4	7.8,18.0	1.7	1.6,1.8	15.6	15.2,16.1	6.0	5.9,6.2

All data adjusted for sampling weights and presented as median with 25^th^,75^th^ percentile or mean with 95% Confidence Interval; geometric mean given for TSH. Tg concentration of 5-7 yr was higher than 11-14 yr (*P* = 0.002), and Tg concentration of 8-10 yr was higher than 11-14 yr (*P* = 0.031). Free T4 concentration of 5-7 yr higher than 11-14 yr (*P* = 0.013). Free T4 concentration of NZEO lower than Māori (*P* = 0.010) and Pacific (*P* = 0.002). Socioeconomic status = SES, New Zealand European and Other Ethnicities = NZEO.

**Table 2:** Effect of sex, age, socioeconomic status and ethnicity on Thyroglobulin (Tg) and TSH concentration

	**Tg**		**TSH**	
	**Ratio (95% Cl)^a^**	**P**	**Ratio (95% Cl)**	**P**
Sex	1.14 (0.96,1.36)	0.134	1.03 (0.96,1.13)	0.366
SES	1.03 (0.97,1.11)	0.415	1.03 (0.98,1.07)	0.270
Age Category	0.86 (0.78,0.93)	0.000	1.02 (0.96,1.08)	0.596
Ethnicity	1.05 (0.98,1.13)	0.142	1.02 (0.99, 1.06)	0.212

^a^Ratio (95% Confidence Interval) of log-transformed data by regression analysis.

Socioeconomic Status = SES.

**Table 3:** Thyroglobulin (Tg), TSH, free T4, and free T3 concentrations by percentile in New Zealand children

	**Percentile**							
	**n**	**2.5**	**5**	**10**	**50**	**90**	**95**	**97.5**
Tg (μg/L)	1153	2.9	4.3	5.4	12.9	28.8	34.5	40.9
5-7 yrs	315	4.0	5.3	6.1	16.2	32.5	40.4	46.7
8-10 yrs	438	2.7	4.3	5.4	12.5	26.7	33.3	33.7
11-14 yrs	400	2.8	3.8	5.0	11.1	26.2	33.7	37.9
TSH (mU/L)	1153	0.7	0.8	1.0	1.7	2.9	3.5	4.1
Free T4 (pmol/L)	1153	11.7	12.2	12.7	15.0	18.2	19.3	20.3
5-7 yrs	315	12.4	12.7	13.2	15.3	18.5	20.0	20.9
8-10 yrs	551	12.0	12.4	12.9	15.2	18.4	19.0	20.3
11-14 yrs	400	11.1	11.6	12.4	14.4	17.2	18.2	19.7
NZEO	333	11.2	12.0	12.4	14.4	17.3	18.5	20.3
Māori	338	11.7	12.4	12.8	15.2	18.4	19.3	20.2
Pacific	482	11.7	12.3	12.9	15.4	18.5	19.5	20.6
Free T3 (pmol/L)	1153	4.9	5.1	5.3	6.0	6.7	7.0	7.1

For each indice, data is presented for entire sample and by category only for those variables shown to be significant in regression analysis. New Zealand European and Other Ethnicities = NZEO.

**Table 4:** Concentration of thyroid indices if urinary iodine concentration (UIC) <50 μg/L or ≥50 μg/L and <100 μg/L or ≥100 μg/L

	**Tg (μg/L)**	**P**	**TSH (mU/L)**	**P**	**Free T4 (pmol/L)**	**P**	**Free T3 (pmol/L)**	**P**
	**Mean (95% Cl)**		**Mean (95% Cl)**		**Mean (95% Cl)**		**Mean (95% Cl)**	
UIC < 50 (μg/L)	15.6 (14.0,17.3)	0.001	1.7 (1.6, 1.9)	0.669	14.7 (14.3,15.2)	0.271	6.1 (6.0,6.2)	0.002
UIC ≥ 50 (μg/L)	12.3 (11.1, 13.5)		1.7 (1.6, 1.8)		15.0 (14.6, 15.3)		5.9 (5.8, 6.0)	
UIC < 100 (μg/L)	13.9 (12.8, 14.9)	0.000	1.7 (1.6, 1.8)	0.236	14.9 (14.5, 15.2)	0.630	6.0 (6.0, 6.1)	0.001
UIC ≥ 100 (μg/L)	10.3 (8.8, 12.1)		1.8 (1.6,1.9)		15.0 (14.5, 15.5)		5.9 (5.8, 6.0)	

Tg and TSH values presented as geometric mean (95% Confidence Interval) and free T4 and T3 values presented as mean (95% Confidence Interval) as determined by regression analysis. Values of 50 μg/L and 100 μg/L based on ICCIDD/UNICEF/WHO [7] recommended cut-offs for UIC. Thyroglobulin = Tg.

The mean (95% CI) fT4 concentration for New Zealand children was 14.9 (14.6‒15.2) pmol/L (Table 1); fT4 concentration did not differ by sex and socioeconomic status (Table 5). There was a significant effect of age, such that increasing age category resulted in a 0.49 pmol/L decrease in fT4 concentration (Table 5). The mean fT4 concentration of children aged 5‒7 yr (15.4 pmol/L) was significantly higher than children aged 11‒14 yr (14.4 pmol/L; *P* = 0.013), but was not different from children aged 8‒10 yr (15.0 pmol/L; *P* = 0.441); there was no difference in the fT4 concentration between children aged 8‒10 yr and 11‒14 yr (*P* = 0.146). There was also a significant effect of ethnicity (*P* = 0.002) (Table 5). NZEO children had a significantly lower mean fT4 concentration (14.6 pmol/L) than Māori children (15.4 pmol/L; *P* = 0.010) or Pacific children (15.6 pmol/L; *P* = 0.002); there was no significant difference between the fT4 concentrations of Māori or Pacific children. The 2.5th percentile of fT4 was highest in children aged 5‒7 yrs but lower in NZEO children compared to Māori or Pacific children (Table 3). The mean (95% Confidence Interval) fT3 concentration for New Zealand children was 6.0 (5.9‒6.1) pmol/L (Table 1); fT3 concentration did not differ by sex, age, socioeconomic status and ethnicity (Table 5). The mean fT3 concentration of children with an UIC <50 μg/L was significantly higher than in children with an UIC ≥50 μg/L (6.1 vs 5.9 pmol/L; *P* = 0.002) (Table 4). The (geometric) mean Tg of children with an UIC <100 μg/L was significantly higher than in children with an UIC ≥100 μg/L (6.0 vs 5.9 pmol/L; *P* = 0.001) (Table 4).

**Table 5:** Effect of sex, age, socioeconomic status and ethnicity on Free T4 and Free T3 concentration

	**Free T4 (pmol/L)**		**Free T3 (pmol/L)**	
	**β (95% Cl)^a^**	**P**	**β (95% Cl)**	**P**
Sex	0.16 (-0.29,0.62)	0.482	0.03 (-0.07,0.14)	0.511
SES	-0.31 (-0.64, 0.03)	0.072	-0.02 (-0.08,0.09)	0.870
Age Category	-0.49 (-0.15, -0.84)	0.004	-02.02 (-0.09,0.05)	0.570
Ethnicity	-0.43 (-0.17,-0.70)	0.002	-0.01 (-0.07,0.07)	0.860

^a^β coefficient (95% Confidence Interval) by regression analysis.

Socioeconomic = SES.

## Discussion

The median UIC of children in this study was 68 μg/L, which falls between 50–99 μg/L, and according to WHO/UNICEF/ICCIDD, indicates that New Zealand children were mildly iodine deficient. Mild iodine deficiency has also been reported in Australian schoolchildren with a median UIC ranging from 74‒143 μg/L [8], and more recently in 14‒15 yr girls from the United Kingdom with a median UIC of 80 μg/L [9]. In a smaller study in New Zealand on a random sample of 300 schoolchildren 8‒10 yr, a similar median UIC of 66 μg/L was found [13]. In this earlier study, blood samples were not obtained from children but thyroid volume was measured by ultrasonography. Using the 1997 cut‒offs for thyroid volume, which were the only available reference data at that time [20], 10% of the children in this smaller study had goiter; when the data were reanalyzed using revised cut‒offs for thyroid volume [21], approximately 30% of the children were classified as having goiter [22]. Although thyroid volume was not measured in the national survey reported here, the similar method of recruitment and median UIC between the earlier study of 8‒10 yr olds and this survey suggests that up to a third of New Zealand children would have had goiter in 2002.

The median Tg concentration of children in this survey of 12.9 μg/L falls within the range of 10.0‒19.9 μg/L, and further supports the results of the urinary analysis that New Zealand children were mildly iodine deficient. In healthy individuals free of thyroid disease, Tg increases with an increase in thyroid volume, thus Tg is an indirect index of goiter. The elevated Tg found in this survey substantiates our view that a large proportion of New Zealand children would have had goiter when the survey was conducted. We found a significant inverse relationship between age and Tg concentration. Djemli et al. [23] also found that Tg concentration decreased with age in children, in contrast to Zimmermann et al. who reported that the difference in Tg with respect to age was small [16]. Of particular interest in this study was the finding that New Zealand children with an UIC ≥100 μg/L had a Tg concentration of 10.3 μg/L, providing evidence that the 10 μg/L cut-off proposed by WHO/UNICEF/ICCIDD for classifying mild iodine deficiency is appropriate for population studies of schoolchildren.

Because of the large intra‒ and inter‒individual variation in UIC [24], a limitation of UIC is that it can only be used to assess the iodine status of a population, but not of the individuals in that population. There is considerable interest, worldwide, in the development of an index of iodine status that can be used to assess iodine deficiency and its severity in individuals. Indeed, compared to other micronutrients such as iron and many vitamins, there is currently no biochemical measure to diagnose mild iodine deficiency in an individual. Soldin et al. used data from NHANES III and found that there was no relationship between UIC and TSH or T4 concentration [25]. Serum Tg concentration holds promise as an index of individual iodine status, but further studies are needed to determine its’ specificity and sensitivity with regard to iodine deficiency. Furthermore, Tg concentration can be affected by a number of factors including interassay variability and the presence of Tg antibodies (Tg‒Ab), which can elevate the concentration of Tg. Zimmermann et al. [16] developed a dried blood spot method for Tg determination, however, to our knowledge, this method is not widely used nor been reproduced in other countries. Regardless of whether Tg can be used to identify iodine deficiency in an individual, our study shows that the median Tg concentration of a group of children can be used as an index of iodine status for a population, and if the dried blood spot method was used, could eliminate the need to collect urine samples under difficult field conditions.

The (geometric) mean TSH concentration of New Zealand schoolchildren of 1.7 mU/L was similar to values published in two other studies of children [23,26] but lower than that reported in a 6–14 year old Austrian hospital‒based pediatric population [27]. In contrast, higher median TSH concentrations were observed in American children 12–19 yr from NHANES III (i.e. girls: 1.3 mU/L and boys: 1.5 mU/L) [28]. Most studies have found that TSH decreased with age, however, we did not observe an age effect in New Zealand schoolchildren. Furthermore, we did not find TSH concentration differed by sex, although both age and sex specific reference ranges for TSH do exist.

The mean fT4 concentration of children in this survey was 14.9 pmol/L and there was a significant effect of both age and ethnicity on fT4. A decrease in fT4 with increasing age has been observed in most other studies of children [23,26,29]. As with TSH, there are small differences in the actual mean fT4 concentration for children obtained from different studies, including our New Zealand data, however, this likely reflects methodological differences with the types of assays used to determine fT4. To our knowledge, this is the first study to find an effect of ethnicity on fT4 concentration, such that Caucasian children (i.e. NZEO) had a lower fT4 than Māori or Pacific children. The mean fT3 concentration of children in this survey was similar to other studies [27,29]. We found no effect of age or sex on fT3 concentration, in agreement with the findings of Soldin et al. [29], however, Kapelari et al. [27] did report a small decline in fT3 concentration with increasing age. In this survey, New Zealand schoolchildren with a low UIC (i.e. either <50 μg/L or <100 μg/L) had significantly higher fT3 concentration than children with a UIC above these cut-offs, however, this difference was only 0.1 pmol/L and is unlikely to have biological significance. The mean fT3, fT4, and TSH concentrations for these children fell within normal reference ranges, which was not unexpected as changes in these indices only occur in moderate to severe iodine ficiency. In studies whose aim is to assess iodine status in schoolchildren, particularly in developed countries where iodine deficiency is unlikely to be severe, only measures of UIC and Tg are required.

This survey is one of the largest recent studies of the iodine status of schoolchildren published to date, but does have limitations. The serum samples had undergone up to four freeze‒thaw cycles prior to analysis, although we found that freeze‒thaw cycles had no effect on either TSH or Tg concentration. We used plasma samples for the determination of fT3 and fT4, which had a recovery >97% for both fT3 and fT4 compared to serum samples from the same individuals. We did not measure Tg‒Ab in our study due to limited financial resources. Zimmermann et al. suggests that screening for Tg‒Ab may not be necessary as a number of studies report a low prevalence (i.e. <5%) of Tg‒Ab in children [16].

## Conclusions

This survey has comprehensively examined the iodine status of a representative sample of schoolchildren using a wide range of biochemical indices. Both the median UIC and Tg fell within the range indicative of mild iodine deficiency as recommended by WHO/UNICEF/ICCIDD; this study shows that both are good population indices of mild iodine deficiency. Little is known about the consequences of mild iodine deficiency in childhood as most research has focused on the effects of moderate and severe iodine deficiency. Given the role of thyroid hormones in the brain, a lack of iodine in childhood could affect the developing brain. A cross-sectional study of Spanish children found that children with an UIC <100 μg/L were at greater risk of having an IQ below 70 [30]. An intervention study conducted in New Zealand of mildly iodine deficient 10‒13 yr old children found that an improvement in iodine status as a result of daily iodine supplementation for seven months also improved aspects of cognition [31]. In response to the widespread mild iodine deficiency observed in the current survey, the New Zealand government introduced the mandatory fortification of bread with iodized salt in late 2009; no other commercial food products are fortified with iodine. An improvement in iodine status of New Zealand children, confirmed by either an increase in median UIC or a decrease in median Tg concentration, has yet to be confirmed.

## Abbreviations

CI: Confidence Interval; CV: coefficient of variation; fT3: free tri­‒iodothyronine; fT4: free thyroxine; ICCIDD: International Committee for the Control of Iodine Deficiency Disorders; NZEO: New Zealand European and Other Ethnicities; Tg: thyroglobulin; Tg­‒Ab: thyroglobulin antibodies; TSH: thyroxine; UIC: urinary iodine concentration; UNICEF: United Nations Children’s Fund; WHO: World Health Organization; yr: year.

## Competing interests

The authors declare they have no competing interests.

## Authors’ contributions

WRP and NW were involved in the planning, implementation and data collection for the Children’s Nutrition Survey. SAS and CDT were involved in the planning of the iodine component of the survey, and supervised the analysis of the blood and urine samples. SAS undertook the statistical analysis. The manuscript was drafted by SAS with contributions from CDT, NW, and WRP. All authors commented on the final draft. All authors read and approved the final manuscript.
